# Intranasal delivery of mitochondrial protein humanin rescues cell death and promotes mitochondrial function in Parkinson's disease

**DOI:** 10.7150/thno.84165

**Published:** 2023-05-29

**Authors:** Kyung Hwa Kim

**Affiliations:** Department of Health Sciences, The Graduate School of Dong-A University, 840 Hadan-dong, Saha-gu, Busan 49315, Korea.

**Keywords:** mitochondria-derived peptide, humanin, Parkinson's disease, mitochondrial biogenesis, autoinduction

## Abstract

**Rationale:** Mitochondrial dysfunction is a key factor in the pathogenesis of Parkinson's disease (PD). Accordingly, many aspects of mitochondrial function have been studied as a putative therapeutic target. Here we present a novel strategy to promote mitochondrial function and protect against Parkinson's disease by the peptide encoded within mitochondrial genome, mitochondria-derived peptide (MDP) humanin (HN).

**Methods:** To test humanin as a potential biomarker in PD, we measured protein levels of circulating humanin from the plasma of PD patients and transgenic or neurotoxic mouse models of PD. Next, we aimed to identify whether HN peptide treatment can regulate its activity or expression. Using mouse models of PD, we assessed HN delivery to the brain via the nasal route of administration. We further revealed a possible mechanism underlying the therapeutic effectiveness of HN peptide for PD using *in vitro* and *ex vivo* model of PD.

**Results:** Although the expression of intracellular HN was not correlated with PD, HN treatment itself could induce intracellular HN expression and enhance mitochondrial biogenesis inducing mitochondrial gene expression. After intranasal administration, HN peptide resulted in neuroprotection and behavioral recovery in an animal model of PD. Interestingly, HN peptide following intranasal delivery was found within the brain, mainly via the trigeminal pathways. Mechanistically, HN treatment induced activation of phosphatidylinositol-3-kinase/protein kinase B (PI3K/AKT) signaling pathway which led to enhanced mitochondrial biogenesis resulting in upregulation of mitochondrial gene including humanin.

**Conclusion**: These data support a novel role of mitochondrial protein humanin in mitochondrial function and neuronal survival against Parkinson's disease, in which humanin treatment is sufficient for stimulating mitochondrial gene expression.

## Introduction

Parkinson's disease (PD) is a common neurodegenerative disease, which has become a serious issue worldwide. Typical clinical features of PD include motor manifestations, such as bradykinesia, resting tremors, and postural instability 1. Although several physiological processes have been implicated in the pathogenesis of PD, some studies have reported the central role of mitochondrial dysfunction in PD 2, 3. Moreover, the etiology of PD is complex, with mitochondrial dysfunction being a common manifestation of sporadic and monogenic PD. In PD, various causes lead to dysfunctional mitochondria, including impaired mitochondrial biogenesis, increased reactive oxygen species (ROS) production, defective mitophagy and mitochondrial dynamics, electron transport chain (ETC) dysfunction, calcium (Ca^2+^) imbalance, and indirect interference with mitochondrial function 2, 4, 5, affecting a range of vital cell functions and causing neuronal cell death 4. A few genes have been elucidated as the monogenic causes of familial PD since the discovery of the first PD-associated gene—α-synuclein (α-Syn)—in 1997 6. Furthermore, mutations in several genes, including α-Syn, leucine-rich repeat kinase 2 (*LRRK2*), Parkin, and *PINK1* genes, are directly linked to mitochondrial dysfunction 7. Recently, new PD- genes, such as coiled-coil-helix-coiled-coil-helix domain-containing 2 (*CHCHD2*) 8 and vacuolar protein sorting-associated protein 35 (*VPS35*) 9, have been discovered and research is in progress to establish a connection between mitochondrial dysfunction and PD pathogenesis. Although mitochondrial DNA (mtDNA) mutations responsible for PD have not been identified, mutations in the genes responsible for mtDNA stability and maintenance, such as polymerase gamma (*POLG*) 10 and mitochondrial replicative helicase (Twinkle) 11, have been associated with neurodegeneration of dopaminergic neurons in PD. These findings suggest the importance of identifying novel mitochondria-associated genes encoded in mtDNA that effectively regulate mitochondrial function for the treatment of PD.

Humanin (HN) is a novel protein encoded by the mitochondrial genome and is known as a mitochondria-derived peptide (MDP). HN was originally identified as an anti-apoptotic peptide; its cDNA was isolated from rescued neuronal cells from patients with Alzheimer's disease (AD) 12. Previous studies have reported the therapeutic potency of HN against several diseases including AD 13, cardiovascular disorders 14, and metabolic disorders 15 by administering synthetic HN or its analog synthetic peptides. Moreover, in humans, HN is localized at multiple sites, including the plasma membrane (bound to cell surface receptors) 16, body fluids such as blood 17, and cytosol 18. As a membrane-bound protein, HN binds to membrane receptors activating several core intracellular signaling pathways, including the Ras-dependent extracellular signal-regulated kinase (ERK) 1/2 and signal transducer and activator of transcription 3 (STAT3) pathways 19. Inside the cell, HN is known to bind to pro-apoptotic molecules, such as insulin-like growth factor binding protein 3 (IGFBP3) 20 and Bcl2-associated X protein (BAX) 18, to inhibit cell apoptosis. Indeed, intracellular HN has been detected in various tissues, including those crucial for metabolic control, such as the liver, muscles, and hypothalamus 21. However, the underlying mechanisms of stimulation on humanin expression are still to be explored.

Given the potential importance of the HN peptide as a therapeutic target, a strategy to improve the expression and activity of humanin is necessary. In this study, synthetic HN peptide displayed an interesting property to activate its own mRNA expression and thereby increase intracellular protein levels. With intranasal route of delivery, HN peptide also gains access to the brain, resulting in significant neuroprotective effects in animal models of PD. Furthermore, we discovered that mitochondrial biogenesis was indispensible for humanin induction and its continued gene expression is necessary for neuroprotective effect of humanin. Promisingly, therapeutic interventions aimed at inducing gene expression of humanin by its own peptide treatment can promote mitochondrial biogenesis and neuroprotection, highlighting an important option for developing an effective treatment strategy against PD.

## Materials and methods

### Animals

C57BL/6-wild type (WT) and C57BL/6-Tg ((NSE-hαSyn)Korl) mice were obtained from Daehan Biolink (Osong, South Korea) and the National Institute of Food and Drug Safety Evaluation (Cheongju, South Korea), respectively. Timed pregnant Sprague-Dawley rats were obtained from Daehan Biolink. All animals were maintained under pathogen-free conditions and a 12-h light/dark cycle at a temperature of 22 ± 3 °C, with food and water provided ad libitum. All animals were handled in accordance with good animal practice, as approved by the Institutional Animal Care and Use Committee (IACUC) of Dong-A University (DIACUC-20-25; Busan, South Korea).

### Human samples

Human plasma samples were collected from patients with sporadic PD. All participants provided written informed consent. The samples and related data were provided by the Biobank of Korea, Chungbuk National University Hospital (CBNUH, Cheongju, South Korea), a member of the Korea Biobank Network. This study was approved by the Institutional Review Board both at CBNUH and Dong-A University. The participants were evaluated and examined by PD specialists at the CBNUH according to the United Kingdom Parkinson's Disease Society Brain Bank criteria. The healthy controls did not have personal or family history of PD. The exclusion criteria for all participants were hematologic malignancies, known pregnancy, and metabolic disorders.

### MPTP challenge in mice

A mouse model of PD was generated using the neurotoxin MPTP-HCl (Sigma-Aldrich, St. Louis, MO, USA), as previously described 22. Seven- to eight-week-old male C57BL/6J mice were injected with MPTP (20 mg/kg/day) for five consecutive days for the subacute delivery of MPTP. The mice in the control group received the same volume of phosphate buffer saline (PBS).

### Primary mesencephalic dopaminergic neuron culture

Primary mesencephalic cell cultures were prepared using gestational day 13.5 embryos from C57BL/6J mice, as previously described 23. The mesencephalon was dissected from the embryos and dissociated in a medium containing 0.25% trypsin-EDTA and 0.05% DNase in PBS for 15 min at 37 °C. Tissue samples were mechanically dissociated by gentle pipetting using Pasteur pipettes. After centrifugation at 400 x *g* for 5 min, the cells were plated in Neurobasal-A medium (Thermo Fisher Scientific, Waltham, MA, USA) supplemented with 2% B27 supplement (Thermo Fisher Scientific), 1× Glutamax (Thermo Fisher Scientific), 5% fetal bovine serum (FBS), 0.36% D-glucose, and 1% Penicillin/Streptomycin (P/S) in poly-L-ornithine/laminin (Sigma-Aldrich)-coated plates. On day 4 *in vitro* (DIV), the plating medium was replaced, and the neurons were used on DIV 8 for subsequent experiments.

### Cell culture and differentiation

The SH-SY5Y human neuroblastoma cell line and PC12 rat pheochromocytoma cell line were purchased from the Korean Cell Line Bank (Seoul, South Korea). Undifferentiated SH-SY5Y cells were grown in Dulbecco's modified Eagle's medium nutrient mixture F-12 (DMEM/F12; Thermo Fisher Scientific) supplemented with 10% FBS and 1% P/S. Undifferentiated PC12 cells were grown in DMEM (Thermo Fisher Scientific) supplemented with 5% FBS, 5% horse serum, and 1% P/S on poly-D-lysine (PDL, Sigma-Aldrich) plates.

For the neuronal differentiation of SH-SY5Y cells, all-trans-retinoic acid (RA; Sigma-Aldrich) was used as previously described, with slight modifications 24. Briefly, the cells were seeded in a minimal essential medium (MEM, Thermo Fisher Scientific) containing 10% FBS, 1% P/S, and glutamax (1×; Thermo Fisher Scientific). After 2 days, the medium was replaced with MEM containing 2% FBS, 1× glutamax, 1% P/S, and 10 μM RA and cultured for another 2 days. For full differentiation, the cells were washed and cultured in Neurobasal medium (Thermo Fisher Scientific) supplemented with B-27 supplement (Thermo Fisher Scientific), 1× glutamax, 1% P/S, and 10 μM RA for 5 and 6 days. The differentiated SH-SY5Y cells were used at 8 days in vitro for subsequent experiments.

For neuronal differentiation of PC12 cells, the cells were grown in DMEM containing 1.5% horse serum, 1.5% FBS, 1% P/S, and 100 ng/mL nerve growth factor (NGF, Thermo Fisher Scientific) every 2 days. PC12 cells were differentiated for 7 days and used for subsequent experiments.

For generating SH-SY5Y Rho^0^ cells depleted of mitochondrial DNA, we treated the cells with ethidium bromide (0.5 μg/ml) (Sigma-Aldrich) for 2 months as described previously 25. SH-SY5Y Rho^0^ cells were grown in the same culture medium but supplemented with an additional 50 μg/ml of uridine and 100 μg/ml of pyruvate to support cell growth.

### Humanin administration

For HN treatment, HN was synthesized as per Fmoc-based SPPS at GL Biochem (Shanghai, China). The lyophilized synthesized HN peptide or HN peptide conjugated with fluorescein isothiocyanate (FITC-HN) was dissolved in Dimethyl sulfoxide (DMSO) and incubated at 37 ℃ with mild stirring (100 rpm) for 1 h to prepare a 10 mM stock solution. The stock solutions were kept at -20 ℃ until use.

For HN treatment of cells, HN stock solution was first incubated at 37 ℃ with mild stirring for 0.5 h. The solution was then added to the culture medium to achieve a desirable final concentration as described above.

For intranasal HN treatment, the synthetic HN peptide was delivered according to a procedure reported by Capsoni *et al* 26. The synthetic HN peptide was diluted in ddH_2_O to a concentration of 0.1 and 5 mg/kg for mice (total volume of 40 μL) at 37 ℃ with mild stirring. The mice were held firmly in one hand in a supine position with their head at 90°. Synthetic HN was administered in the form of 5-μL drops over 20 min in alternating nostrils every 3 min. After administration, the heads of the mice were held in the same position to prevent loss of the applied solution from the nares.

### Rat brain organotypic slice culture

Postnatal 5-7-day-old rat pups were used to prepare organotypic slices, as previously described, with minor modifications 27. The brain of anesthetized pups was aseptically removed. The brain hemispheres were separated and cut sagittally into 350-μm slices using a MacIlwain tissue chopper (MacIlwain, Mickle Laboratory, Cambridge, UK). The slices were transferred to ice-cold Gray's Balanced Salt solution containing CaCl_2_ (1.5 mM), NaCl (136 mM), KCl (5 mM), KH_2_PO_4_ (0.2 mM), MgCl_2_ (1 mM), MgSO_4_ (0.2 mM), NaHCO_3_ (0.2 mM), Na_2_PO_4_ (0.08 mM), and glucose (5.5 mM). The slices were placed on a membrane insert (Millicell, Bedford, MA, USA) in a 6-well plate with 1 mL of culture medium containing 50% MEM, 25% HBSS (Hank's balanced salt solution), 25% horse serum, 6.5 mg/mL glucose, 2 mM L-glutamine, 1 x glutamax, 1% P/S and 1% amphotericin B (all from Thermo Fisher Scientific) below the insert. The medium was changed on the first day after culture, then every 2 days, and was maintained up to DIV 7 for subsequent experiments.

### Immunocytochemistry and immunofluorescence analysis

For staining, the cells were seeded on PDL-coated glass slides and treated as indicated above. The samples were fixed using methanol and permeabilized using 0.1% Triton X-100 in PBS. After blocking with 5% bovine serum albumin (BSA), the cells were incubated with the appropriate primary antibody [anti-HN (1:500, Cat no # RA19000, Neuromics, Edina, MN, USA), anti-TH (1:500, Cat no # P40101, Pel-Freez Biologicals, Rogers, AR, USA), anti-lectin (1:200, Cat no # L32482, Thermo Fisher Scientific), anti-α-SMA (1:500, Cat no # ab5694, Abcam, Cambridge, MA, USA), and anti-GFAP (1:200, Cat no # sc-33673, Santa Cruz, Santa Cruz, USA)]. After incubation, the samples were washed and incubated with Alexa Fluor 594- or 488-conjugated antibodies (Thermo Fisher Scientific). The nuclei were stained using Hoechst 33342 (Sigma-Aldrich) and mounted using an anti-fade solution (Thermo Fisher Scientific). Fluorescent images were captured at the Neuroscience Translational Research Solution Center (Busan, South Korea) using an LSM 700 confocal microscope (Zeiss, Jena, Germany). The mitochondrial network morphology was assessed using the FIJI/ImageJ software as described previously 28.

To stain the mouse brain tissue samples, transcardial perfusion was performed using PBS and 4% paraformaldehyde in PBS. The brain and trigeminal ganglia were removed, post-fixed using 4% paraformaldehyde in PBS, and stored in a 30% sucrose solution at 4 ℃ until they sank. Coronal sections were prepared (thickness: 10 µm) using a sliding microtome. After blocking with 5% BSA, the samples were incubated with the appropriate primary antibody as mentioned above. After incubation with the primary antibodies, the tissue samples were incubated with Alexa Fluor 594- or 488-conjugated antibodies (Thermo Fisher Scientific) for immunofluorescence or with secondary biotinylated anti-rabbit IgG (H+L) (Vector Laboratories, Burlingame, CA, USA) for immunohistochemistry. To enhance immune signals in immunohistochemistry, a Vectastain Elite ABC Standard Kit (Vector Laboratories) was used, as per the manufacturer's instructions. The stained sections were scanned using a Pannormic MIDI scanner (3DHISTECH, Budapest, Hungary) at the Neuroscience Translational Research Solution Center. Unbiased stereological analyses were performed as previously described 22. For immunofluorescence analysis, the nuclei were stained using Hoechst 33342 (Sigma-Aldrich) and mounted using an anti-fade solution (Thermo Fisher Scientific). Fluorescent images were captured at the Neuroscience Translational Research Solution Center (Busan, South Korea) using an LSM 700 confocal microscope (Zeiss, Jena, Germany). Mitochondrial network morphology was further assessed using the FIJI/ImageJ software as described previously 28.

### Western blot analysis

Mouse brain tissue samples and cells were homogenized in radioimmunoprecipitation assay (RIPA) buffer (Cell Signaling, Danvers, MA, USA) in the presence of a protease and phosphatase inhibitor cocktail (Thermo Fisher Scientific). The samples were sonicated on ice, and the supernatant was collected after centrifugation at 4°C at 10,000 g for 5 min. The protein samples were loaded and separated using Tris-glycine SDS-PAGE. To detect the HN peptide, the proteins were separated into three layers of tricine SDS-PAGE, as previously described 29. The separated proteins were transferred to nitrocellulose membranes (GenDEPOT, Barker, TX, USA). The membranes were blocked for 0.5 h, followed by incubation with appropriate primary antibodies overnight at 4°C. The primary antibodies used were as follows: anti-tyrosine hydroxylase (Cat # P40101, Pel-Freez Biologicals), anti-HN (Cat # RA19000, Neuromics), anti-β-actin (Cat # GTX109639, GeneTex, Irvine, CA, USA), and anti-GAPDH (Cat # AM4300, Thermo Fisher Scientific). After washing, the membranes were incubated with horseradish peroxidase-labeled secondary antibodies (Cell Signaling Technology) and visualized using enhanced chemiluminescence reagents (DoGen, Seoul, South Korea). The results were analyzed using the ImageJ densitometry system. In order to detect circulating humanin in human plasma samples, proteins were extracted by trichloroacetic acid (TCA) precipitation 30. Loading of an equal amount of protein was confirmed by Coomassie staining of plasma samples which were depleted of albumin by Minute™ Albumin Depletion Reagent (Invent Biotechnologies, Plymouth, MN, USA).

### Enzyme-Linked Immunosorbent Assay (ELISA)

A commercially available ELISA kit (MyBioSource, San Diego, CA, USA) was used to measure plasma and brain HN levels. To measure the protein levels of HN, brain homogenates (100 μg protein/well) and 100 μL of plasma samples were loaded onto competitive ELISA plates according to the manufacturer's instructions. The absorbance was measured using a microplate reader (Molecular Devices, Sunnyvale, CA, USA) at a wavelength of 450 nm.

### Locomotor activity analysis

To assess bradykinesia in the MPTP-treated mice, a pole test was performed as previously described 31. The mice were placed facing upward on top of a vertical pole at an approximate height of 30 cm. The time taken to face downward and the total time taken to descend the pole were recorded. The mice completed the task thrice, and the average of these trials was calculated.

### Transfection assay

We constructed a plasmid encoding HN by subcloning the HN-encoding gene into pEGFP-C1 vector. The full-length HN construct was obtained by gene synthesis (Bioneer, Daejeon, South Korea) and was subcloned at the BglII and SalI sites of pEGFP-C1. The differentiated PC12 cells were transiently transfected with the vector using Lipofectamine 3000 (Thermo Fisher Scientific) according to the manufacturer's protocol.

### PCR analysis of mitochondrial DNA

The loss of mtDNA in SH-SY5Y Rho^0^ cells was determined by standard PCR with three pairs of primers (Table S1), two specifically for mtDNA (mt-ND4, humanin) and one specifically for nuclear DNA (ATP5PB). PCR amplicon bands from all 5 putative Rho^0^ clones displayed a loss of mtDNA in 10% polyacrylamide gels. Assessment of the brightness of the bands was conducted by using the Image J program (http://rsb.info.nih.gov/ij/).

### Measurement of mitochondrial ROS levels

The SH-SY5Y cells were cultured in 96-well plates for 24 h before treatment. At the indicated time points, the cellular medium was removed, and the cells were washed with PBS. Mitochondrial ROS levels were measured using MitoSOX Red (Thermo Fisher Scientific) as described previously 32. The cells were counterstained using Hoechst 33342 (Sigma-Aldrich). Fluorescence intensity was quantified using a Synergy H1 multimode microplate reader (BioTek, Winooski, VT, USA) according to the manufacturer's protocol.

### Mitochondrial membrane potential

The cells were seeded in 96-well plates and treated as described above. To analyze the mitochondrial membrane potential, TMRM (Thermo Fisher Scientific) was used according to the manufacturer's instructions. The cells were counterstained using Hoechst 33342 (Sigma-Aldrich). Fluorescence intensity was assessed using a Synergy H1 multimode microplate reader or an LSR Fortessa cytometer at the Neuroscience Translational Research Solution Center.

### Cell viability assay

Following treatment with the aforementioned reagents, the cells were washed and treated with 3-(4,5-dimethylthiazol-2-yl)-2,5-diphenyl-tetrazolium bromide (MTT, Thermo Fisher Scientific). Briefly, the cells were seeded in a 96-well plate at a density of 5 × 10^3^ cells/well. After 24 h, a neurotoxin 1-methyl-4-phenylpyridinium (MPP^+^) (Sigma-Aldrich) was applied to induce toxicity in neuronal cells. Next, the cells were incubated with MTT for 4 h, and formazan crystals were solubilized using DMSO. Absorbance was measured using a multi-plate reader at a wavelength of 570 nm, and the viability of the cells was recorded as a percentage of the control.

### ATP level measurement

The organotypic slice cultures were exposed to 6-OHDA at the tissue surface on DIV 8 to avoid apoptosis due to nutrients and to induce proper maturation. After treatment with 6-OHDA (100 μM) for 2 h, the slice cultures were collected from the inserts in 6-well culture plates and washed with PBS. After centrifugation at 400 x *g* for 5 min, the slices were resuspended in the culture medium of organotypic slices and seeded in 96-well at 5 × 10^3^ cells/well. Intracellular ATP was quantified using the CellTiter-Glo Luminescent Viability Kit (Promega Corporation, Madison, WI, USA), according to the manufacturer's protocol. Luminescence was measured using a SpectraMax L Microplate Reader (Molecular Devices).

### Quantitative real-time polymerase chain reaction (Real-time qPCR)

In order to demonstrate the impact of HN peptide on mitochondrial transcripts, we purchased a first-in-class specific inhibitor of mitochondrial transcription inositol 4-methyltransferase (IMT1) from MedChemExpress (Monmouth Junction, NJ, USA) and co-treated with HN peptide for 24 h in SH-SY5Y cells. For real-time qPCR, total RNA was extracted using TRIzol reagent (Thermo Fisher Scientific). cDNA was synthesized using a cDNA synthesis kit (Bioneer Corporation), according to the manufacturer's protocol. The mRNA levels were analyzed in a LightCycler 96 (Roche, Basel, Switzerland) using SYBR Green I master mix and SensiFAST™ SYBR^®^ No-ROX Kit (Bioline, Paris, France). B2M (beta-2-microglobulin) gene expression was used to normalize gene expression data. The primer sequences used in this experiment are in Table S2.

### Statistical analysis

Statistical analyses were performed using the GraphPad Prism software (San Diego, CA, USA). Two-tailed unpaired t-tests were used for comparisons between the two groups. Group differences were assessed using one-way or two-way analysis of variance (ANOVA) for multiple groups, followed by multiple comparison tests. *P* < 0.05 was considered statistically significant.

## Results

### Intracellular expression of humanin is not correlated with PD

**Figure 1:**
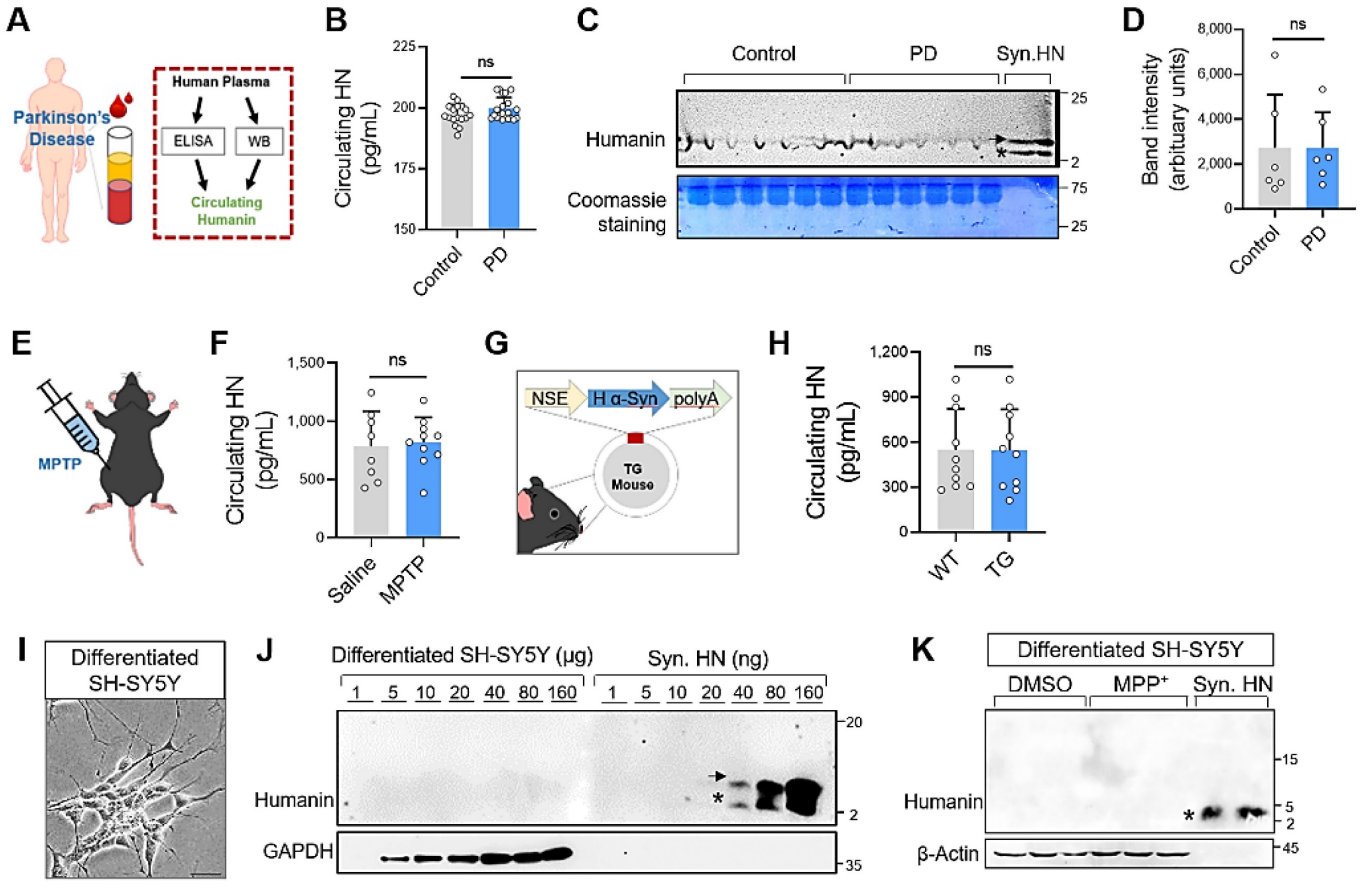
** Humanin protein levels are not correlate with Parkinson's disease. (A)** Schematic of the study setup. **(B, C, D)** Circulating levels of humanin in the plasma of PD patients using ELISA **(B)** (*n* = 21 PD patients, *n* = 17 healthy control groups) and western blot analysis **(C, D)** (*n* = 6 per group). Circulating humanin in the plasma shows weak expression both in PD and control groups. Synthetic humanin peptide (Syn. HN: 100 ng). Asterisk and arrow indicate the monomeric (~3 kDa) and dimeric form (~ 6 kDa) of humanin, respectively. **(E, F)** Mouse model of PD treated with MPTP (20 mg/kg/day, ip) for 5 consecutive days. Two days after the final MPTP treatment, circulating humanin was measured in the plasma of MPTP-treated mice using ELISA. **(G, H)** Transgenic (TG) mouse models of PD overexpressing human α-synuclein gene (α-syn). Using ELISA, circulating humanin was assessed in the plasma of transgenic PD mice at the age of 10 - 12 months. **(I)** Phase-contrast pictures of differentiated SH-SY5Y cells. Scale bar, 20 μm. **(J)** Quantitative WB analysis of cell lysates from differentiated SH-SY5Y cells (1 - 160 μg) and synthetic humanin (HN) peptide (1 - 160 ng) by tricine-SDS-PAGE. **(K)** No clear alteration of intracellular humanin in SH-SY5Y cells against neurotoxin MPP^+^ exposure. Synthetic humanin peptide (Syn. HN: 100 ng). Data are expressed as mean ± SEM (standard error of the mean). NS: not significant; *P* > 0.05. WT: Wild-type.

Here, we hypothesized that humanin could be a prognostic biomarker for Parkinson's disease. To evaluate this hypothesis, we examined the circulating HN levels in patients with PD (Figure 1A). The demographic and clinical information of all participants is summarized in Table S1. Using enzyme-linked immunosorbent assay (ELISA), we detected approximately 197 pg/mL HN in the plasma of healthy controls; unexpectedly, a significant difference in the circulating HN levels was not observed in the PD group (Figure 1B). Western blot analysis also displayed lowly expressed circulating humanin from plasma both in the PD and control groups (Figure 1C, D). There was no significant difference between groups.

To confirm this no relationship between humanin expression and PD, we measured circulating humanin in a mouse model of PD using methyl-4-phenyl-1,2,3,6-tetrahydropyridine (MPTP) intoxication (Figure 1E). Consistent with the results obtained using human samples, a significant difference was not observed in the circulating HN levels between MPTP-induced PD mice and saline-treated mice (Figure 1F). Moreover, we also found that HN levels were unchanged in an α-Syn-overexpressing transgenic PD mouse model, as per ELISA (Figure 1G, H).

We next attempted to assess intracellular expression level of humanin using an *in vitro* model (Figure 1I). By applying a quantitative Western blot analysis, we could detect clear bands of humanin within the range of loading (> 40 ng) of synthetic HN peptides. However, intracellular humanin was barely detectable even after loading large amounts of proteins (≤ 160 μg) from differentiated SH-SY5Y human neuroblastoma cells. Collectively, our findings indicate that circulating humanin is not altered with PD and intracellular humanin is relatively low expressed or hardly detected in neuronal cells, suggesting that humanin might not be a potential biomarker for PD.

### HN treatment induces intracellular humanin expression in neuronal cell

**Figure 2:**
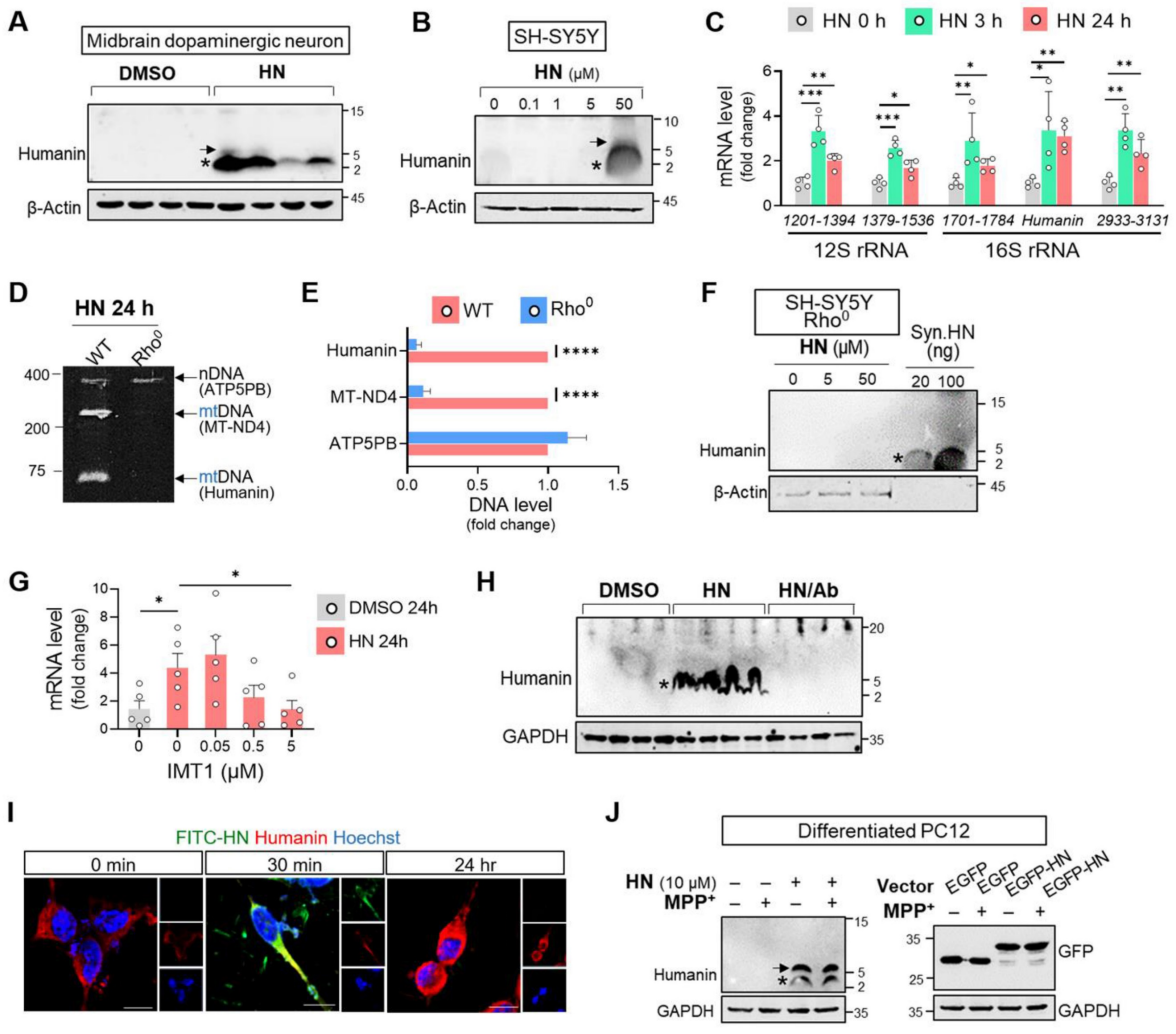
** Humanin treatment induces intracellular humanin expression in neuronal cells. (A, B)** HN treatment for 24 h results in upregulation of intracellular humanin in mouse midbrain dopaminergic neurons **(A) (**1 μM) and SH-SY5Y cells **(B)** in dose-dependent manner**.** Asterisk and arrow indicate the monomeric and dimeric forms of humanin, respectively. **(C)** Real-time qPCR analysis amplifying different regions of the mitochondrial rRNAs (12S rRNA and 16S rRNA) after HN treatment for designated hours. Each value on the x-axis represents a targeted sequence within mitochondrial genome. **(D, E)** Conventional PCR with specific primers targeting nuclear DNA (nDNA; ATP5PB) and mitochondrial DNA (mt-ND4 or humanin) from Rho^0^ cells devoid of mtDNA. The bands on polyacrylamide gels were quantified. **(F)** Western blot analysis assessing intracellular humanin levels in SH-SY5Y Rho^0^ cells after HN treatment (20 μM) for 24 h. Synthetic humanin peptide (Syn. HN: 100 ng). **(G)** Real-time qPCR analysis by co-treating IMT1 inhibiting mitochondrial gene expression with HN in SH-SY5Y cells. **(H)** Western blot analysis with treating HN peptide (20 μM) with HN antibody (HN/Ab) in SH-SY5Y cells. HN/Ab mixture (3 times excess peptide to antibody by weight). **(I)** Immunofluorescence of fluorophores conjugated HN (FITC-HN: green) in SH-SY5Y cells for indicated hours. Intracellular humanin was stained (red). Scale bar, 10 μm. **(J)** Western blot of upregulated intracellular humanin by HN peptide or transfection with EGFP-tagged humanin expression plasmid (EGFP-HN) in differentiated PC12 cells. EGFP plasmid (EGFP). Data are expressed as means ± SEM. (*P < 0.05; **P < 0.01; ***P < 0.001; ****P < 0.0001). HN: humanin.

Considering huge therapeutic potentials of humanin treatment against many neurodegenerative diseases 33, we next hypothesized that humanin peptide treatment could stimulate its activity or expression. As shown in Figure 2, basal humanin was undetectable in midbrain dopaminergic neurons from embryonic mouse (Figure 2A) and SH-SY5Y cells (Figure 2B). However, HN synthethic treatment for 24 h induced its intracellular expression in a dose-dependent manner displaying the putative homo-oligomerization of humanin. We further monitored the transcript levels of humanin in mitochondrial ribosomal RNA (rRNA) following HN treatment. Within 3 h of HN treatment, we interestingly observed significant upregulation of different regions across rRNA as well as humanin. The levels of stimulated mitochondrial transcripts induced by HN treatment slightly declined over time but were significantly upregulated up to 24 h following treatment. These results suggested the possibility that HN treatment itself stimulates mitochondrial gene expression.

However, this induction effect by the HN peptide was abolished in mtDNA-depleted (Rho**^0^**) SH-SY5Y cells, as demonstrated by conventional PCR (Figures 2D, E) and western blot analysis (Figure 2F). To validate the stimulating role of humanin on mitochondrial transcripts, we suppressed the production of mitochondrial double-stranded RNAs (mt-RNAs) with IMT1, a small molecule inhibitor recently reported by Bonekamp et al. 34. As shown in Figure 2G, IMT1 co-treatment in SH-SY5Y cells markedly attenuated humanin transcripts induced by HN peptide treatment in a dose-dependent manner. Furthermore, antibody to HN blocked humanin induction demonstrating a unique HN-dependent induction of humanin (Figure 2H). To discriminate exogenously added HN peptide and intracellular humanin, we treated FITC-conjugated humanin (FITC-HN) in SH-SY5Y cells. As shown in Figure 2I, we observed a weak humanin signal in SH-SY5Y cells. Within 30 min of FITC-HN treatment, SH-SY5Y cells had a strong intracellular fluorescence signal while no detectable fluorescent signal after 24 h suggesting degradation of the FITC-HN peptide. However, 24 h-treated cells had a strong signal of intracellular humanin.

Furthermore, we examined whether HN treatment induces intracellular humanin expression even under the pathological conditions of PD. As shown in Figure 2J, we also observed that HN treatment upregulated intracellular humanin in differentiated PC12 cells exposed to MPP^+^. These effects of inducing expression were further confirmed with an enhanced green fluorescent protein (EGFP)-tagged HN transfection. Therefore, synthetic HN-induced humanin in the neuronal cells may mainly act by promoting mitochondrial gene expression.

### HN treatment protects neurons in both animal and cellular models of PD

**Figure 3:**
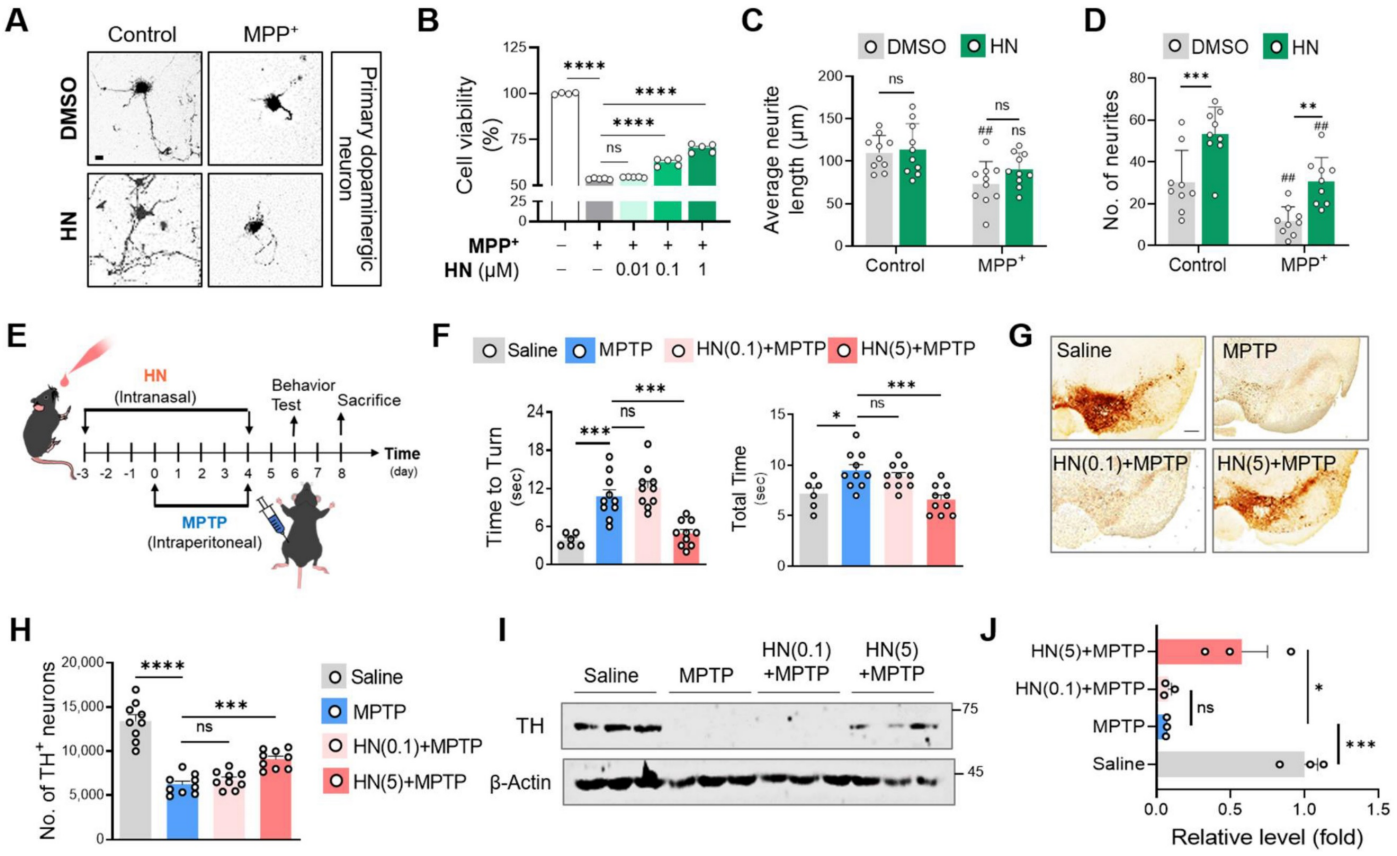
** Humanin treatment protects neurons and ameliorates behavioral defects in PD. (A, B, C, D)** Murine primary dopaminergic neurons were pre-treated with DMSO or HN (1 μM) for 12 h and then challenged with MPP^+^ (100 μM) for 24 h. **(A)** Photomicrographs show a protective effect of humanin. Scale bar, 10 μm. **(B)** Viability of primary dopaminergic neurons using MTT assay. Neurite length **(C)** and neurite number** (D)** were decreased after MPP^+^ exposure (^#^*P* < 0.05; ^##^*P* < 0.01; control versus MPP^+^ group). These damages were recovered by pre-treatment with HN peptide. **(E)** Schematic diagram of experimental schedule following intranasal injection of HN (0.1 mg/kg, 5 mg/kg) in MPTP-challenged mice at indicated time points. **(F)** Behavioral evaluation with pole test. **(G)** Representative images of tyrosine hydroxylase (TH) staining from substantia nigra of indicated groups. Scale bar, 100 μm. **(H)** Stereotaxic assessment of TH-positive neurons. **(I)** Western blot images of TH proteins from the striatum brain lysates of the indicated groups. **(J)** Three independent Western blots quantified by densitometry using Image Lab Software. Data are expressed as means ± SEM. (ns, *P* > 0.05; *P < 0.05; **P < 0.01; ***P < 0.001; ****P < 0.0001). HN: humanin.

Based on our results that HN treatment stimulates mitochondrial gene expression, we hypothesized that HN administration might have neuroprotective effects against PD. As shown in Figure 3, we observed that MPP^+^ exposure caused dopamine neuron death in primary cultures of the mouse mesencephalon (Figures 3A, B) as well as morphological abnormalities, including neurite loss (Figures 3C, D). In contrast, HN treatment rescued the neurite loss and neuronal death induced by MPP^+^ in a dose-dependent manner. Moreover, HN treatment also increased neurite number, suggesting that HN enhances neurite outgrowth. The average length of all sprouting neurites per neuron was slightly higher in the HN-treated neurons than that of the DMSO-treated group; however, the difference was not statistically significant.

Next, we tested the therapeutic impact of HN using the MPTP mouse model of PD. To explore therapeutic HN peptide targeting brain tissues, we injected HN peptide intranasally in PD mice (Figure 3E). Clearly, intranasal administration of HN in PD mice led to steady motor improvements in a dose-dependent manner (Figure 3F). Brain tissue samples stained using tyrosine hydroxylase (TH) exhibited significant loss of dopaminergic neurons in the substantia nigra pars compacta of MPTP-lesioned mice (Figure 3G, H), whereas intranasal treatment with HN significantly restored TH expression in PD mice in a dose-dependent manner. Western blot analysis confirmed the impact of intranasal delivery of HN on protecting TH^+^ dopaminergic neurons in PD mice (Figure 3I, J). These findings suggest that HN inhibits and protects dopaminergic neurons from PD and improves motor deficits in PD.

### HN can be delivered to the brain through trigeminal pathways

**Figure 4:**
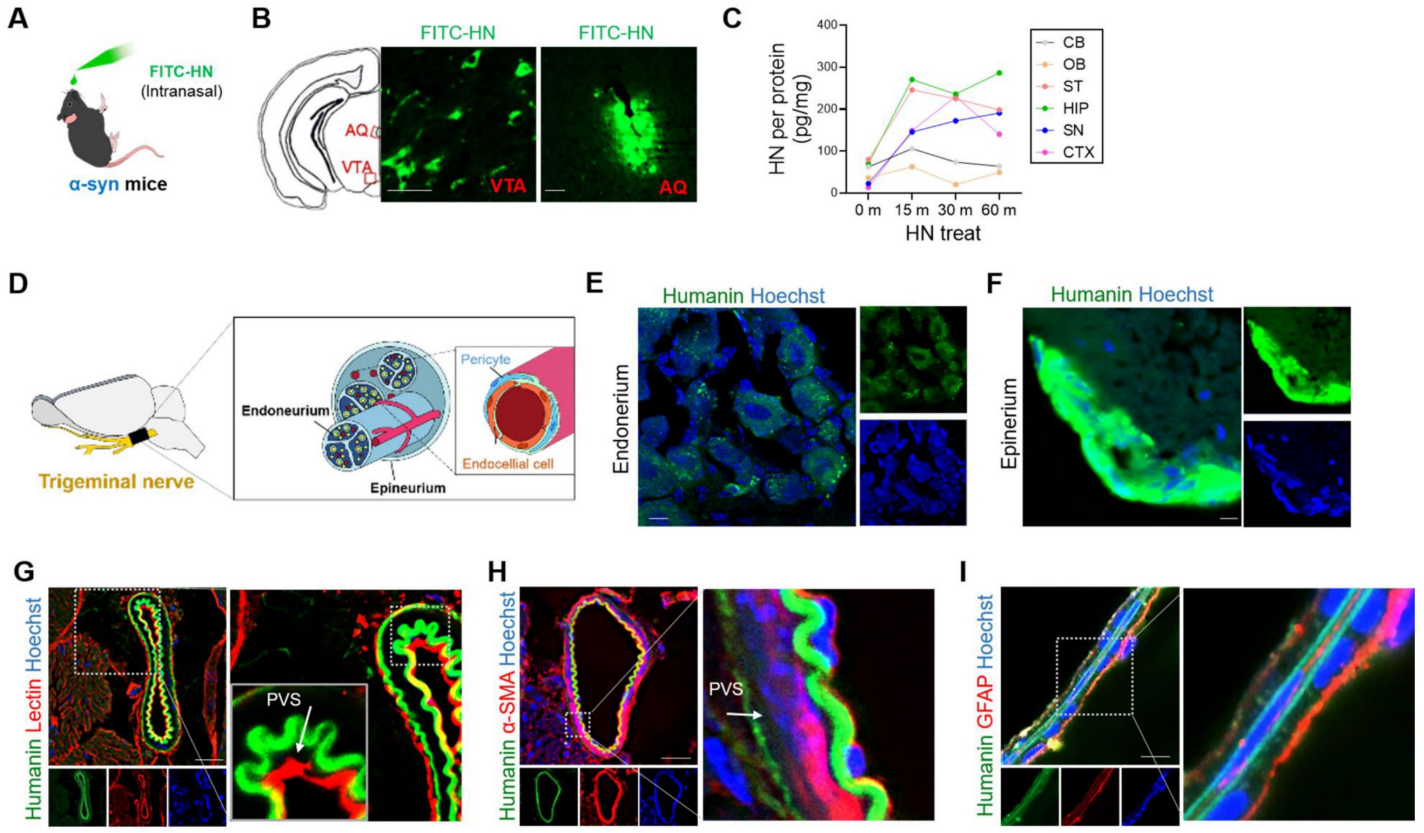
** Intranasally administered humanin can reach the brain via the trigeminal nerve along the perivascular and perineural spaces. (A)** Schematic diagram of experiments performed after FITC-tagged humanin (FITC-HN) treatment of transgenic PD mice overexpressing human α-syn.** (B)** FITC-HN was detected in the midbrain regions including the ventral tegmental nerve (VTA) and cerebral aqueduct (AQ) within 30 m after intranasal administration. Scale bar, 50 μm. **(C)** ELISA for detecting FITC-HN in the brain tissue samples after intranasal administration at the indicated time points. CB, cerebellum; OB, olfactory bulb; ST, striatum; HIP, hippocampus; SN, substantia nigra; CTX, cerebral cortex. **(D)** Putative delivery route of FITC-HN distribution after intranasal injection to the brain through the trigeminal nerve. **(E, F, G, H)** Within 10 minutes after intranasal treatment, FITC-HN was detected associated with the epineurium **(E)** and, to a lesser extent, the endoneurium **(F)** of the trigeminal nerves as well as in the perivascular space (PVS), as evaluated by staining blood vessels with tomato lectin **(G)** and alpha smooth actin (α-SMA) **(H)**. **(I)** Within 30 minutes after intranasal treatment, FITC-HN for brain was found in microvascular endothelial cells surrounded by astrocytic endfeet, stained with anti-glial fibrillary acidic protein (GFAP). Scale bar, 10 μm. HN: humanin.

Here, we explored whether intranasal administration allows direct delivery of HN peptide to the brain. Thus, we intranasally administered fluorescently labeled HN peptide (FITC-HN) to human α-Syn-overexpressing transgenic PD mice (Figure 4A). Within 30 min of injection, FITC-HN was clearly detectable in the midbrain region surrounding the ventral tegmental area (VTA) and aqueduct (AQ) (Figure 4B). Interestingly, intranasally-delivered HN exhibited widespread brain distribution, lasted for up to 1 h after intranasal administration of HN (Figure 4C).

Next, we aimed to demonstrate the potential brain-entry routes of intranasally administered HN. The olfactory and trigeminal routes are the main pathways for the entry of an intranasally administered drug into the brain 35. Thus, we examined the cross-sections of the trigeminal nerves after intranasal administration of FITC-HN in transgenic PD mice (Figure 4D). Within 30 minutes after nasal administrateion, we observed FITC-HN distribution of diffuse to punctate in the perineural space of the endoneurium, a compartment located between nerve axons (Figure 4E). A more prominent FITC-HN signal was detected in the perineurium region, which is a space in the outermost layer of the nerve (Figure 4F). We hypothesized that the perivascular spaces (PVS) associated with the trigeminal nerves may provide potential pathways for the distribution of HN in the brain following intranasal administration. To determine whether HN utilizes the vascular pathway, we imaged FITC-HN in the blood vessels associated with the trigeminal nerves by specifically staining with tomato lectin to label endothelial cells (Figure 4G) and alpha smooth actin (α-SMA) to label perivascular mural cells including pericytes (Figure 4H). These results suggest that intranasally-administered HN may be mainly transported through arteries of trigeminal nerves, partially from entering the PVS of blood vessels of the nose. In brain parenchyma, FITC-HN was seen closely surrounding microvascular endothelial cells lining the brain microvessels of BBB as determined by astrocyte end feet labeling with an anti-glial fibrillary acidic protein (GFAP) (Figure 4I). Thus, our results indicate that intranasally administered HN is delivered to the brain tissues through the trigeminal pathway via blood vessels.

### HN treatment enhances mitochondrial biogenesis and dynamics

**Figure 5:**
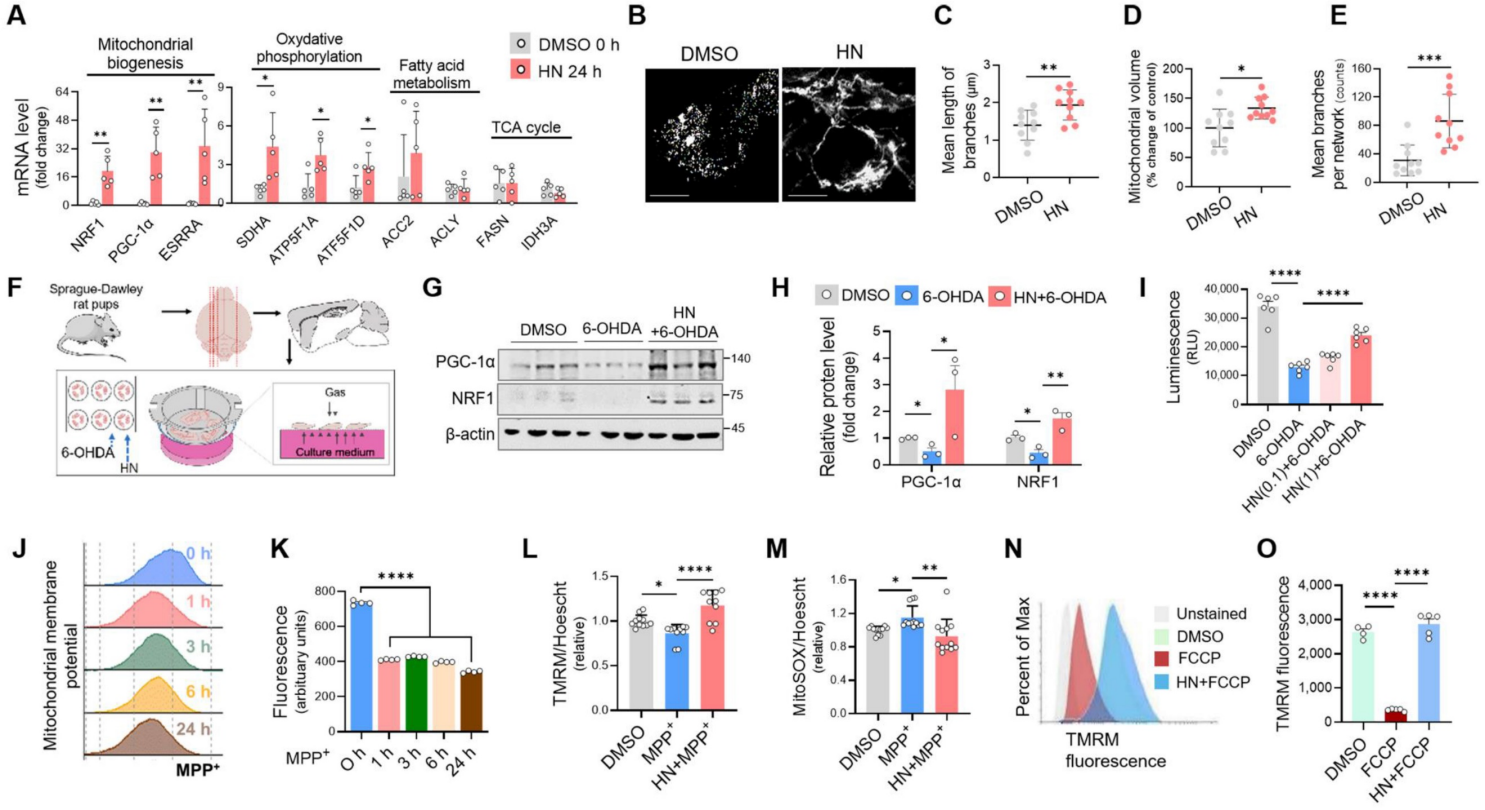
** Humanin treatment exhibits enhanced mitochondrial biogenesis, restoring mitochondrial impairment in Parkinson's disease. (A)** Real-time qPCR analysis showing expression of genes associated with mitochondrial biogenesis (*NRF1*, *PGC-1α*, and *ESRRA*), oxidative phosphorylation (*SDHA*, *ATF5F1A*, and *ATF5F1D*), fatty acid metabolism (*ACC2* and *ACLY*), and TCA cycle (*FASN* and *IDH3A*) in SH-SY5Y cells after HN treatment (20 μM, 24 h). **(B, C, D, E)** Representative images **(B)** of stained mitochondria with MitoTracker Green in SH-SY5Y cells after HN treatment (20 μM, 24 h). The branch length **(C)**, mitochondrial volume **(D)**, and the number of branches **(E)** were calculated for analyzing the mitochondrial network. Scale bar, 10 μm. **(F)** Schematic diagram of experimental setup using *ex vivo* model of PD. The rat organotypic slices were pre-treated with HN (1 μM, 24 h) and then exposed with neurotoxin 6-OHDA (100 μM, 2 h). **(G)** Western blot analysis for proteins involved in mitochondrial biogenesis from *ex vivo* organotypic culture slices as indicated groups.** (I)** ATP determination based on luminescent signal proportion to the amount of ATP from *ex vivo* organotypic culture slices with humanin pre-treatment (24 h; 0.1 μM or 1 μM) and 6-OHDA exposure. **(J, K)** Dramatic loss of mitochondrial membrane potential stained with TMRM (100 nM) in SH-SY5Y cells following MPP^+^ exposure for indicated times. **(L, M)** Recovered mitochondrial membrane potential **(L)** and reduced mitochondrial superoxide production **(M)** by pre-treatment with HN in MPP^+^-challenged SH-SY5Y cells assessing with flow cytometry. The raw fluorescence for each probe was normalized against Hoechst 33342 (1 μg/ml) fluorescence to control for any variation in cell number. **(N, O)** Reversed mitochondrial membrane depolarization induced by FCCP (5 μM, 15 min) by HN pre-treatment (24 h; 20 μM) in SH-SY5Y cells. Data are expressed as means ± SEM. (*P < 0.05; **P < 0.01; ***P < 0.001; ****P < 0.0001). HN: humanin.

Our recent study revealed a novel function of another MDP mitochondrial open reading frame of the 12S rRNA-c (MOTS-c) 36 that controls the expression of a nuclear gene responsible for the maintenance of cellular fitness in response to injury 32. Here, we investigated whether HN could alter the expression profiles of genes involved in mitochondrial function using real-time qPCR. As shown in Figure 5A, HN treatment resulted in a significant increase in the transcript levels of the genes associated with mitochondrial biogenesis (*PGC-1α*, *NRF1*, and estrogen-related receptor alpha (*ESRRA*) and oxidative phosphorylation (succinate dehydrogenase complex flavoprotein subunit A (*SDHA*), ATP synthase F1 subunit alpha (*ATP5F1A*), and ATP synthase F1 subunit delta (*ATP5F1D*)) in SH-SY5Y cells. However, a significant alteration was not observed in the expression of the genes encoding proteins associated with fatty acid metabolism and the tricarboxylic acid cycle (TCA) cycle.

Next, we investigated whether HN treatment can strongly activate mitochondrial function. After HN peptide treatment in SH-SY5Y cells, increased mitochondrial mass was displayed, as determined by MitoTracker Green staining (Figure 5B). Moreover, HN treatment resulted in longer rods and branches of mitochondria (Figure 5C) and an increase in mitochondrial volume (Figure 5D) and the number of branches per mitochondrial network (Figure 5E) compared to those of DMSO-treated cells. These results suggest that HN treatment boosts mitochondrial biogenesis and affects the mitochondrial network morphology. Next, we also validated the therapeutic impact of HN on mitochondrial function using an *ex vivo* model of PD utilizing organotypic brain cultures (Figure 5F-I). Western blot analysis of markers of mitochondrial biogenesis, such as *PGC-1α* and *NRF1*, revealed that HN pre-treatment prior to 6-OHDA stimulated mitochondrial biogenesis (Figure 5G, H). Moreover, HN pre-treatment effectively inhibited significant depletion of ATP levels induced by 6-OHDA in organotypic slices (Figure 5I). Considering the role of mitochondrial damage in PD pathogenesis 37, we further examined mitochondrial function by monitoring changes in the mitochondrial membrane potential using tetramethylrhodamine methyl ester (TMRM) staining in MPP^+^-challenged SH-SY5Y cells. Following MPP^+^ exposure, a mitochondrial membrane potential was a rapid fall (30 min) (Figure 5J, K). This collapsed potential lasted up to 24 h when significant cell death was detected following MPP^+^ treatment. However, pre-treatment with HN before MPP^+^ treatment effectively inhibited reduction in mitochondrial potential (Figure 5L) and mitochondrial ROS production (Figure 5M) caused by neurotoxin MPP^+^. Furthermore, HN pre-treatment was effective in restoring carbonylcyanide-p-trifluoromethoxyphenylhydrazone (FCCP)-induced mitochondrial depolarization in SH-SY5Y cells, as assessed by flow cytometry (Figure 5N, O). Collectively, these results suggest that HN peptide displays benefits in PD treatment by restoring mitochondrial function.

### Neuroprotective effect of HN treatment is PI3K/AKT signaling pathway-dependent

**Figure 6:**
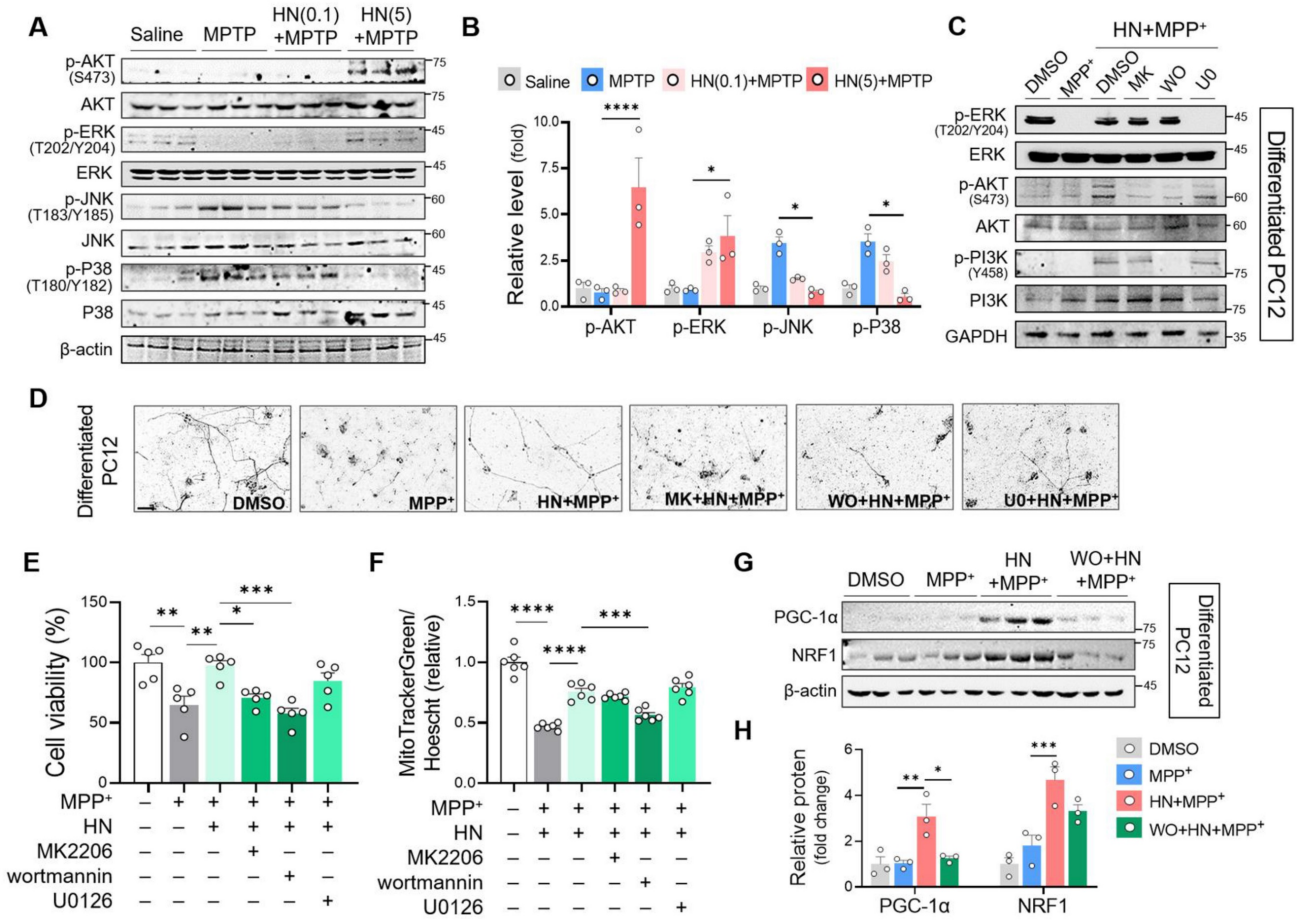
** Recovery of mitochondrial biogenesis and neuroprotection by humanin treatment is PI3K/AKT signaling-dependent. (A, B)** Western blot analysis to measure PI3K/AKT, ERK, and JNK activity from the striatum brain tissues of MPTP-treated PD mice with HN treatment (0.1 mg/kg, 5 mg/kg) as described above. **(C)** Differentiated PC12 cells were pre-treated with MK2206 (AKT inhibitor, 1 μM), wortmannin (PI3K inhibitor, 1 μM), or U0126 (ERK inhibitor, 10 μM) 2 h before treatment with humanin (HN, 10 μM) prior to MPP^+^ challenge for 24 h (1 mM). Drug effects on these cellular signaling pathways were confirmed by western blot analysis. **(D, E, F)** Phase-contrast pictures **(D)**, MTT assay **(E)**, and mitochondrial mass evaluation **(F)** of differentiated PC12 cells with pre-treatment of MK2206, wortmannin, and U0126 in HN treated MPP^+^-challenged cells.** (G, H)** Western blot analysis of the genes involved in mitochondrial biogenesis with pre-treatment of wormannin in humanin treated MPP^+^-challenged differentiated PC12 cells. Data are expressed as means ± SEM. (*P < 0.05; **P < 0.01; ***P < 0.001; ****P < 0.0001). HN: humanin. MK: MK2206, WO: wortmannin, U0: U0126.

Previous studies have reported the central role of misregulated signaling cascade kinases, including extracellular signal-regulated kinases (ERK), p38 mitogen-activated protein (MAP) kinase, phosphatidylinositol 3‑kinase (PI3K)/protein kinase B (AKT), and c-Jun N-terminal kinase (JNK), in the pathogenesis of PD 38, 39. Here, we aimed to characterize the association between HN and these signaling pathways. As shown in Figures 6A and B, we observed significant activation of JNK and p38 MAPK in the striatum of the PD mice. In contrast, HN treatment inhibited the activation of JNK and p38 MAPK in PD mice. Moreover, we noticed that HN promoted the activation of AKT and ERK in the brain tissue samples of PD mice in a dose-dependent manner. To investigate the mechanism underlying the effects of HN treatment, we used specific inhibitors that target these pathways in differentiated PC12 cells (Figure 6C). Pre-treatment of differentiated PC12 cells with wortmannin (an inhibitor of the PI3K pathway) or MK-2206 (an inhibitor of the AKT pathway) abolished the neuroprotective effect of HN against MPP^+^-induced neurotoxicity (Figures 6D, E). However, pre-treatment with U0126 (an inhibitor of the ERK pathway) did not alter cell viability following HN treatment in MPP^+^-exposed differentiated PC12 cells. Among these inhibitors, wortmannin significantly blocked the impact of HN on enhancing mitochondrial mass (Figure 6F) and promoting mitochondrial biogenesis (Figure 6G, H) in differentiated PC12 cells exposed to MPP^+^. We also observed that pre-treatment with wortmannin blocked the induction of HN expression after HN treatment (Figure S1), indicating a PI3K-dependent mechanism of HN peptide action for inducing its expression. Therefore, these results revealed that exogenous HN could enhance its bioactivity and therapeutic potential through the activation of the PI3K/AKT pathway in PD.

## Discussion

An already large and rapidly growing body of evidence supports the critical role of mitochondrial dysfunction in the pathophysiology of Parkinson's disease 4. Generally, PD-associated mitochondrial dysfunction is defined as reduced mitochondrial biogenesis, diminished membrane potential, and oxidative stress 40. Defective mitochondrial activity in disparate areas will likely lead to harmful effects that link by feedback and feedforward mechanism 2. The initial defects might be mild but could lead to severe dysfunction due to the highly interconnected feature of mitochondria. Probably, these different insults might converge on initiating and activating mitochondrial apoptotic pathways in dopaminergic neurons. Luckily, these complexities of mitochondrial pathways have provided us with an opportunity for drug development by alleviating mitochondrial dysfunction 4. In particular, strategies aimed at promoting mitochondrial biogenesis are attractive for designing effective drugs to treat PD mainly due to their central and complex role to contribute to both the mitochondrial and nuclear genomes that thus crosstalk between the nucleus and mitochondria 41.

In the current study, we target mitochondrial impairment in PD with MDP HN treatment in cellular and animal models of PD. We found that HN treatment stimulated mitochondrial biogenesis, resulting in the induction of expression of HN gene encoded within the mitochondrial genome. Mitochondrial dysfunction was observed prior to cell death in MPP^+^-challenged neuronal cells, suggesting that the impairment of mitochondrial function is involved in the pathogenesis of neurodegeneration in PD 42. Mechanistically, we demonstrated the neuroprotective effect of HN treatment in *in vivo*, *in vitro*, and *ex vivo* PD models, which promotes mitochondrial biogenesis mainly via the PI3K/AKT signaling pathway. We also elucidated that HN entered the brain mainly through the PVS of the trigeminal nerve, similar to the drugs that bypass the BBB 43. Therefore, intranasal delivery of HN promotes mitochondrial function, stimulates its own gene expression, and exhibits a beneficial impact on neurodegeneration, thus it can be used for anti-PD therapy.

Peptides are increasingly being recognized as functional players in key biological cellular processes 44, 45. Recent breakthroughs in analytical and synthetic technologies have significantly accelerated the development of peptide drugs. Since 2000, 33 non-insulin peptide drugs have been approved 46 and more than 170 peptides have been evaluated in clinical trials for a wide range of diseases 47. Nevertheless, the development of peptide drugs for the brain is difficult. The delivery of peptide drugs is generally limited by their poor bioavailability, rapid elimination by the liver, and the BBB 48. Although many new strategies are being explored, little provides a satisfactory efficacy and safety balance for brain delivery. Currently, most approaches for drug delivery into the brain or cerebrospinal fluid are highly risky, invasive, and local.

In contrast, intranasal delivery is an efficient method for the delivery of drugs to the brain 49. Intranasal administration of insulin improves cognitive deficits in patients with AD 50. Using an animal model of AD, Lochhead *et al.* clearly demonstrated that insulin reaches the brain along the trigeminal route, mainly through the PVS, after intranasal administration 51, which is in accordance with the results of the present study. Therefore, this may be the first study to report that intranasal administration of HN can induce wide distribution of HN in the brain targeting Parkinson's disease. Within 10 min of intranasal administration, HN was detected in the PVS and perineural space of the trigeminal nerve, suggesting that the trigeminal pathway may be involved in the delivery of HN to the CNS (Figure 4). Moreover, intranasally administered HN exhibited widespread distribution in the brain within 30 min of a single injection. Although the mechanisms governing transport into the brain remain unknown, PVS of the blood vessels is the potential extracellular pathways that can facilitate rapid and widespread delivery of intranasally administered therapeutics to the brain 43. Previous studies on potential drug therapeutics, including insulin insulin-like growth factor I (IGF-I) 52, vascular endothelial growth factor (VEGF) 53, and transforming growth factor-β1 (TGF-β1) 54, have proposed that intranasally administered compounds can effectively enter the PVS of arteries in the nasal lamina propria that are connected with the arteries of the trigeminal nerves or olfactory bulbs. In accordance with the results of these studies, we observed that FITC-labelled HN was present in the PVS of cerebral arteries and vessels in the brain parenchyma after intranasal administration. HN contains the highly lipophilic region L^9^L^10^L^11^, which is essential for HN secretion. We propose that all characteristics of HN peptides, including lipophilicity, charge, molecular weight, and receptor binding, may contribute to their transport to the brain. In this study, we used a synthetic HN peptide, which was synthesized using the Fmoc solid-phase peptide synthesis strategy (Fmoc SPPS) by elongating the deprotection of the active HN peptide, as described previously 55. Consistent with previous studies 56, we observed that self-dimerization of intracellular HN was upregulated by HN treatment as per tricine-SDS-PAGE. Although the mechanistic details are partially understood, dimerization is essential for the protective function of HN 56. It is plausible that in response to HN binding, the putative HN receptor undergoes oligomerization to initiate downstream signaling. Hashimoto *et al*. suggested that the therapeutic function of HN is mediated by a specific tyrosine kinase system 12. Delineating humanin as a BBB-permeable bioactive peptide and a ligand of the tyrosine kinase receptor will require further work.

Humanin is the first peptide representing a novel group of naturally occurring bioactive peptides encoded by the mitochondrial genome, termed MDPs. Since its discovery in 2001 12, humanin has been proposed as a potential protective peptide for neurological disorders, cardiovascular diseases, and metabolic dysfunction 19. The central role of humanin in the protective response has been extensively studied in AD 13. Maftei *et al*., using affinity mass spectrometry, identified that humanin can directly bind to amyloid β (Aβ), which subsequently disrupts the oligomerization of Aβ 57. Furthermore, S14G-HN administration, a highly potent human derivative, significantly reduced the total amyloid plaque burden in the cerebellum 58, hippocampus, and cortex 59 without altering Aβ production in a transgenic mouse model of AD. Moreover, HN is considered a mitokine 60, a new class of mitochondria-derived signals that act in an endocrine manner. Once secreted, humanin binds to the following two types of receptors: tripartite receptor complex comprising CNTF receptor/WSX-1/gp130 61 and formylpeptide receptor like-1 (FPRL-1) 16. The binding of HN to these receptors activates downstream signaling cascades such as the PI3K/AKT, ERK1/2, and STAT3 pathway 62. Consistent with these previous reports, we observed that humanin was localized in the plasma membrane immediately after HN treatment (Figure 2), suggesting that circulating humanin may induce the activation of multiple intracellular signaling cascades.

With improvements in sequencing technologies, recent comprehensive studies revealed that the humanin mitochondrial genome possesses several functional classes of small open reading frames (sORFs) 19, 63. To date, millions of sORFs of 100 codons or fewer have been discovered in eukaryotic genomes 64, 65. Accumulating evidence exists for the actual translation of these transcribed sORFs which results in the production of a biologically active peptide 63. Humanin is considered the first sORFs encoded from mtDNA 19, 63. Although the precise mechanism of transcription and translation of humanin remains unclear, nascent humanin may be first transcribed as polycistronic RNA from sORFs of 16S rRNA and post-transcriptionally modified into shorter adenylated transcripts 66, 67. Humanin appears to be expressed in a variety of tissues with tissue-specific gene profiles. It is highly expressed in heart, kidney, testis, and skeletal muscles, but less or undetectable in brain 17, 68. Interestingly, humanin presents both intracellularly and in the extracellular space 69. However, little is known about the mechanism of controlling tissue-specific expression signatures and by which intracellular humanin releases or entry into the cells. In our study, intracellular humanin was undetectable in neuronal cells across all the species we estimated. As shown in Fig 2J, a detection limit was about 40 ng of synthetic humanin peptide in the presence of its corresponding primary antibody. On the contrary, we could not quantitatively measure intracellular humanin in a range of 1 - 160 μg of total cell lysates from WB analysis, indicating the need of a precise method to reflect the absolute expression levels.

Here, we investigated whether HN treatment influences its own expression in the cell. HN treatment can upregulate intracellular expression of humanin in part through the induction of mitochondrial biogenesis. After HN treatment, a rapid increase in the transcripts within mitochondrial rRNA was induced in 3 h and was maintained for up to 24 h after HN treatment (Figure 2). We believe that exogenously treated humanin peptide promotes mitochondrial biogenesis, in particular by activating the PI3K/AKT signaling pathway, which results in the stimulation of mitochondrial gene expression. Understanding unexplored features of smORF transcription and translation within the mitochondrial genome is urgently needed.

Therefore, we elucidated that exogenously treated HN peptide protects against PD by enhancing mitochondrial biogenesis, resulting in stimulating expression of mitochondrial genes including humanin. Thus, boosting mitochondrial function with HN peptide may be a promising therapeutic approach against PD.

## Supplementary Material

Supplementary figure and tables.

## Abbreviations

PD: Parkinson's disease
MDP: mitochondria-derived peptide
HN: humanin
ROS: reactive oxygen species
ETC: electron transport chain
Ca^2+^: calcium
α-Syn: α-synuclein
LRRK2: leucine-rich repeat kinase 2
CHCHD2: coiled-coil-helix-coiled-coil-helix domain-containing 2
VPS35: vacuolar protein sorting-associated protein 35
POLG: polymerase gamma
mtDNA: mitochondrial DNA
Twinkle: mitochondrial replicative helicase
AD: Alzheimer's disease
ERK: extracellular signal-regulated kinase
IGFBP3: insulin-like growth factor binding protein 3
BAX: Bcl2-associated X protein
PI3K/AKT: phosphatidylinositol-3-kinase/protein kinase
MOTS-c: mitochondrial open reading frame of the 12S rRNA-c
ESRRA: estrogen-related receptor alpha
SDHA: succinate dehydrogenase complex flavoprotein subunit A
ATP5F1A: ATP synthase F1 subunit alpha
ATP5F1D: ATP synthase F1 subunit delta
TCA: tricarboxylic acid cycle (TCA)
JNK: Jun N-terminal kinase
IGF-1: insulin insulin-like growth factor 1
VEGF: vascular endothelial growth factor
TGF-β1: transforming growth factor-β1
ATP5PB: ATP synthase peripheral stalk-membrane subunit b
MT-ND4: mitochondrially encoded NADH dehydrogenase 4
Aβ: amyloid β
FPRL-1: formylpeptide receptor like-1
tricine-SDS-PAGE: tricine-sodium dodecyl sulfate-polyacrylamide gel electrophoresis
MPP^+^: 1-methyl-4-phenylpyridinium
MPTP: methyl-4-phenyl-1,2,3,6-tetrahydropyridine
rRNA: ribosomal rna
FITC-HN: fluorescein isothiocyanate-conjugated humanin
PVS: perivascular spaces
TH: tyrosine hydroxylase
sORFs: small open reading frames
