# Supplementary material for: Intranasal delivery of mitochondrial protein humanin rescues cell death and promotes mitochondrial function in Parkinson's disease
Source: Theranostics. 2023 May 29;13(10):3330–45. doi: 10.7150/thno.84165 (PMC10283052; doi:10.7150/thno.84165)
Supplement: Supplementary file 1 — Supplementary figure and tables. [file thnov13p3330s1.pdf]

# Intranasal delivery of mitochondrial protein humanin rescues cell death and promotes mitochondrial function in Parkinson's disease

Kyung Hwa Kim

## Supplementary figures and tables

**Table S1. General characteristics of healthy controls and patients with Parkinson’s disease**.

| | PD (n = 21) | HC (n = 17) | p-value |
|---|---|---|---|
| Age (y) | 61.48 ± 8.87 | 58.35 ± 4.05 | 0.19 |
| Gender, male (%) | 76.47 | 57.14 | 0.22 |
| BMI | 24.87 ± 2.51 | 25.70 ± 2.05 | 0.33 |
| UPDRS III (n) | 37.33 (3) | – | – |
| Family history (%) | 19.05 | – | – |

BMI, body mass index; UPDRS III, part III of the Movement Disorders Society Unified Parkinson’s Disease rating scale

**Table S2. Primers used for Real-time qPCR analysis**

| Gene symbol | Forward primer | Reverse primer |
|---|---|---|
| 12S rRNA (1201-1394) | AGCCTGTTCTGTAATCGATAAACC | GCTATCGTAGTTTTCTGGGGTAGA |
| 12S rRNA (1379-1536) | AGAAAACTACGATAGCCCTTATGAAA | TAGGGGTTTTAGTTAAATGTCCTTTG |
| 16S rRNA (1701-1784) | TACTACCAGACAACCTTAGCCAAAC | TACTATATCTATTGCGCCAGGTTTC |
| humanin | TTATGCCCGCCTCTTCACGGGCA | CACGAGGGTTCAGCTGTCTCTTACTTTTA |
| 16S rRNA (2933-3131) | GGATAACAGCGCAATCCTATTC | CTTTCGTACAGGGAGGAATTTG |
| B2M | TGCTGTCTCCATGTTTGATGTATCT | TCTCTGCTCCCCACCTCTAAGT |
| NRF1 | ACAGCAGTGGCAAGATCTCA | GCAAGGCTGTAGTTGGTGCT |
| PGC-1α | CAGAGAACAGAAACAGCAGCA | TGGGGTCAGAGGAAGAGATAAA |
| ESRRA | GAGATCACCAAGCGGAGACG | ATGAGACACCAGTGCATTCAC |
| SDHA | GAGATGTGGTGTCTCGGTCCAT | GCTGTCTCTGAAATGCCAGGCA |
| ATP5F1A | CATCTTGGTCCTGCCCTGAC | GCTGCAGATAGACAAGGCGA |
| ATP5F1D | GTCGTGGTGCATGCAGAG | GAAGAGTCGGCGTTCACTG |
| ACC2 | CAAGTGCAAGATCTGTTTCCCTGATCG | CCGGAAGTTTAGGGTTTTCTGAAGCAT |
| ACLY | TGCTCGATTATGCACTGGAAGT | ATGAACCCCATACTCCTTCCCAG |
| FASN | GGTCTTGAGAGATGGCTTGC | AATTGGCAAAGCCGTAGTTG |
| IDH3A | CCCGCGTGGATCTCTAAGG | AATTTCTGGGCCAATACCATCTC |
| MT-ND4 | CTAGGCTCACTAAACATTCTA | CCTAGTTTTAAGAGTACTGCG |
| ATP5PB | CATCTTGGTCCTGCCCTGAC | GCTGCAGATAGACAAGGCGA |

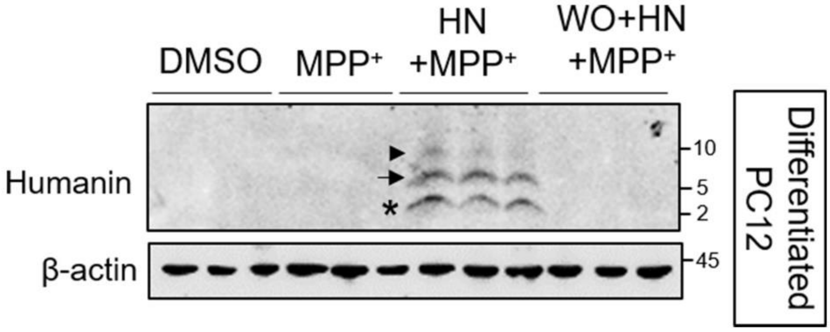


**Figure S1. Upregulation of intracellular humanin induced by humanin treatment is PI3K signaling-dependent.** Western blot analysis assessing the impact of PI3K inhibitor wortmannin on upregulation of intracellular humanin induced by HN treatment (10 μM, 24 h) in $MPP^{+}$-exposed differentiated PC12 cells. Pre-treatment with wortmannin (WO, 1 μM) for 1 h significantly inhibited induction of intracellular humanin by its treatment. Asterisk, arrow, or arrowhead indicates the putative monomeric, dimeric, or trimeric form of humanin, respectively. HN: humanin.
